# Multi Ray Model for Near-Ground Millimeter Wave Radar

**DOI:** 10.3390/s17091983

**Published:** 2017-08-30

**Authors:** Ariel Etinger, Boris Litvak, Yosef Pinhasi

**Affiliations:** Faculty of Engineering, Ariel University, Ariel 40700, Israel; etinger7@ariel.ac.il (A.E.); borisl@ariel.ac.il (B.L.)

**Keywords:** radar, millimeter wave radars, multipath, multi-ray model

## Abstract

A quasi-optical multi-ray model for a short-range millimeter wave radar is presented. The model considers multi-path effects emerging while multiple rays are scattered from the target and reflected to the radar receiver. Among the examined scenarios, the special case of grazing ground reflections is analyzed. Such a case becomes relevant when short range anti-collision radars are employed in vehicles. Such radars operate at millimeter wavelengths, and are aimed at the detection of targets located several tens of meters from the transmitter. Reflections from the road are expected to play a role in the received signal strength, together with the direct line-of-sight beams illuminated and scattered from the target. The model is demonstrated experimentally using radar operating in the W-band. Controlled measurements were done to distinguish between several scattering target features. The experimental setup was designed to imitate vehicle near-ground millimeter wave radars operating in vehicles. A comparison between analytical calculations and experimental results is made and discussed.

## 1. Introduction

Wireless communication systems and radars are developing towards the utilization of millimeter wavelengths above 30 GHz. One of the most important issues at these frequencies is maintaining a proper link with minimal interference and clutter. Millimeter waves (MMW) face disruptive effects including atmospheric attenuation due to weather conditions, fog and rain [1], and losses while passing through objects. In this article, we will focus on the interference caused by the transmitted signal itself due to multi-scattered reflections from the target, as in MMW radars. Such interference results in constructive and destructive summations of the reflected signals, which are space-frequency dependent. The well-known two-ray model is practical for describing this phenomenon in wireless communication links [2,3,4], where the transmitter and the receiver are located at different sites. However, it fails to address other scenarios, such as when the link is at millimeter wavelengths [5,6], operating particularly in urban areas [7,8], or where multiple reflections from objects and buildings are involved. Multipath also occurs in radar links, even if the transmitter and receiver are located in the same place. In addition to the line-of-sight (LOS) wave reflected from the target, scattering due to the ground results in additional paths that interfere with the direct LOS received wave. 

Nowadays, short-range millimeter wave radars operating at 77 GHz are used commercially in anti-collision systems in vehicles [9]. The radar is positioned at the front of the vehicle and gives an alert when the distances to obstacles are too short. In this mode of operation, the radar height above ground is such that not only the direct line of sight (LOS) path exists, but ground-reflected rays are also involved. It is interesting to note that this problem has been addressed with regard to millimeter wave communication links between vehicles, and was investigated using the two-ray model [10,11]. 

In this article, the emergence of multipath signals is investigated while the millimeter wave radar is operating close to the ground. The effect of multiple rays scattered from the target and reflected back to the receiver is analyzed. Several scenarios are studied, revealing different fading of the received signals. The theory is examined using an experimental setup operating in the W band.

Our analysis is based on a multi-ray model, in which waves scattered by the target and reflected by the ground simultaneously arrive at the radar receiver. In MMW radars, where directional antennas are employed, quasi-optical ray analysis is found to be sufficient to describe the multipath phenomena. The resulting model enables one to identify and differ between possible multi-path scenarios, which are also demonstrated experimentally. 

## 2. Millimeter Wave Surface Reflectivity

The reflection of an electromagnetic wave from a surface is characterized by the reflectivity:

(1) $$\rho_{0}=\left|\rho_{0}\right|\cdot e^{j∠\rho_{0}}$$

where ${\left|\rho_{0}\right|}^{2}$ is the power reflectivity and $∠\rho_{0}$ is the phase shift due to the reflection. This is a frequency-dependent complex quantity determined by the dielectric constant *ε′* of the surface, its electrical conductivity *σ*, the wave incident angle *θ*_0_, and its polarization [2]. Table 1 summarizes the dielectric constants of various types of ground at millimeter wave frequencies [12,13,14].

**Figure 1:**
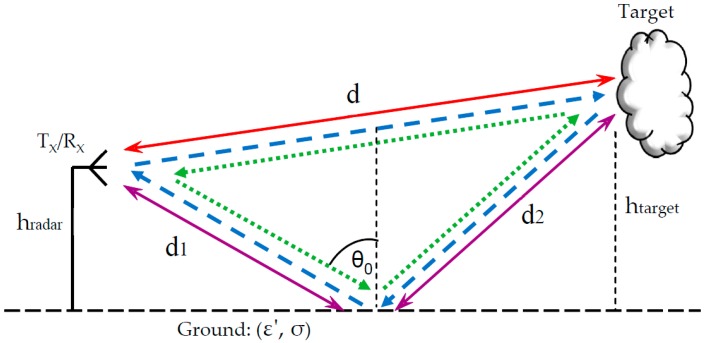
Four main propagation paths in a radar scenario.

Considering reflections from the ground, we define the horizontal polarization as the transverse electric mode (TE), and the vertical polarization as the tangential magnetic field (TM). Figure 1 illustrates four possible paths for radar wave propagation in which reflections from the ground are involved. The reflectivity of the ground is calculated using Fresnel’s equations after introducing the Snell law [2], resulting in:

(2) $$\rho_{0}=\{\begin{array}{cc} \frac{\eta \cos\left(\theta_{0}\right)-\eta_{0}\sqrt{1-\frac{1}{\varepsilon \prime }\sin^{2}\left(\theta_{0}\right)}}{\eta \cos\left(\theta_{0}\right)+\eta_{0}\sqrt{1-\frac{1}{\varepsilon \prime }\sin^{2}\left(\theta_{0}\right)}} & TE\\ \frac{\eta \sqrt{1-\frac{1}{\varepsilon \prime }\sin^{2}\left(\theta_{0}\right)}-\eta_{0}\cos\left(\theta_{0}\right)}{\eta \sqrt{1-\frac{1}{\varepsilon \prime }\sin^{2}\left(\theta_{0}\right)}+\eta_{0}\cos\left(\theta_{0}\right)} & TM \end{array}$$

Assuming that the magnetic permeability of the medium (air and ground) is identical to that of the vacuum $\mu_{0}=4\pi \cdot 10^{-7}\;H/m$, the impedance of the surface is $\eta =\sqrt{\mu_{0}/\left(\varepsilon \prime \varepsilon_{0}\right)}$. Consequently, the expression for the field reflectivity for both polarizations can be simplified to:

(3) $$\rho_{0}=\{\begin{array}{cc} \begin{array}{l} \frac{\cos\left(\theta_{0}\right)-\sqrt{\varepsilon \prime -\sin^{2}\left(\theta_{0}\right)}}{\cos\left(\theta_{0}\right)+\sqrt{\varepsilon \prime -\sin^{2}\left(\theta_{0}\right)}} \end{array} & \begin{array}{l} TE \end{array}\\ \frac{\sqrt{\varepsilon \prime -\sin^{2}\left(\theta_{0}\right)}-\varepsilon \prime \cos\left(\theta_{0}\right)}{\sqrt{\varepsilon \prime -\sin^{2}\left(\theta_{0}\right)}+\varepsilon \prime \cos\left(\theta_{0}\right)} & TM \end{array}$$

**Figure 2:**
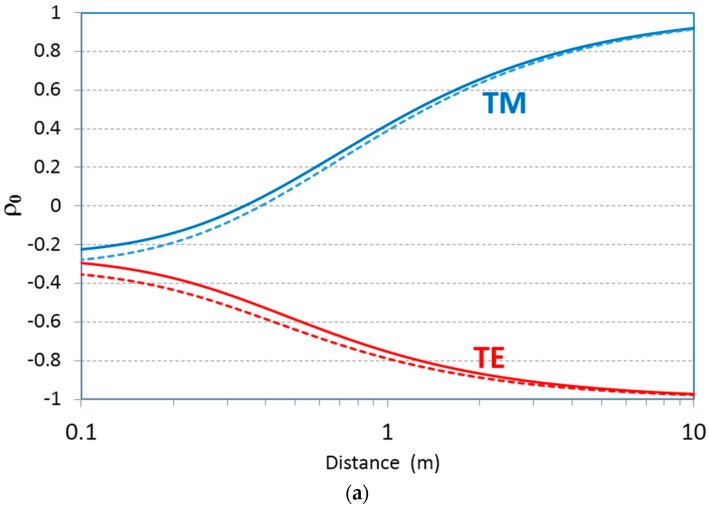

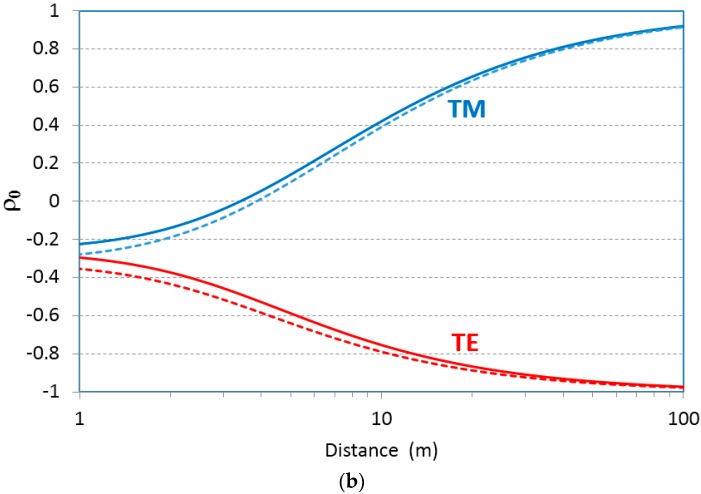
Soil reflectivity as a function of distance between the transmitter and receiver (**a**) when *h_t_ = h_r_ =* 10 cm; and (**b**) when *h_t_ = h_r_ =* 1 cm at frequency 94 GHz. Solid line—little moisture. Dashed line—moist soil.

Figure 2 describes the millimeter wave (94 GHz) reflectivity as a function of range when both the transmitter and receiver are at identical heights *h_t_ = h_r_ =* 10 cm (Figure 2a) and *h_t_ = h_r_ =* 1 cm (Figure 2b) above ground. Graphs are drawn for both polarizations, while two types of soil are considered; soil with low moisture (*ε*′ = 2.9), and moist soil (*ε*′ = 3.7).

## 3. Four-Ray Model for Near-Ground Radar

Considering ground reflections, there are, in general, four possible propagation paths between the transmitter and the receiver, as shown in Figure 1. The number of paths is determined by the scattering characteristics of the target. All four paths exist if the target demonstrates a diffuse reflection, where the incident wave is reflected in many directions due to surface roughness scattering. Such a phenomenon emerges if the reflecting surface irregularities are comparable with radiation wavelength [15]. A Lambertian reflectance is one example, where the scattered wave presents equal brightness from all directions that lie in the half-space adjacent to the surface. The reflections are also at many angles if the target has a rounded shape (as in the bumper of a car).

**Figure 3:**
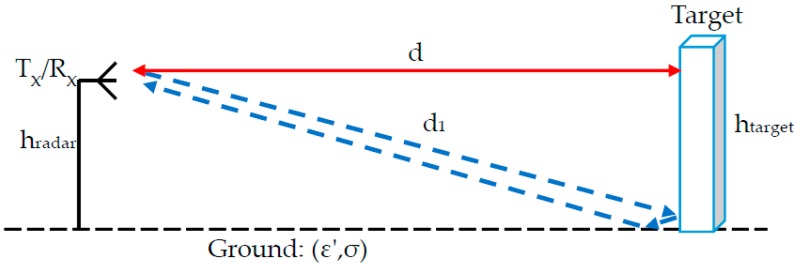
Two main radar propagation paths from flat target close to ground.

In the case of a flat smooth target, the reflection is specular; the angle of reflection equals that of the incident angle. The flat target perpendicular to the ground creates a ‘corner reflector’, resulting in two possible paths, as illustrated in Figure 3.

The total signal received by the radar is a result of the summation of fields scattered from the target and arriving at the receiver via four paths. One path is the line of sight, the direct path. The other three involve reflections from the ground. Assuming that the reflectivity from the target is represented by $\rho_{\mathrm{target}}$, and does not depend on the incident angle, the total field resulting from all four rays is given by the sum:

(4) $$\begin{array}{l} {\tilde{E}}_{\mathrm{Total}}=\underset{Path1}{\underset{︸}{\frac{1}{2d}\cdot e^{-j\frac{2\pi f}{c}\left(2d\right)}\cdot \rho_{\mathrm{target}}}}+\underset{Path2}{\underset{︸}{\frac{1}{d+d_{1}+d_{2}}\cdot e^{-j\frac{2\pi f}{c}\left(d+d_{1}+d_{2}\right)}\cdot \rho_{\mathrm{target}}\cdot \rho_{\mathrm{ground}}}}\\ +\underset{Path3}{\underset{︸}{\frac{1}{d+d_{1}+d_{2}}\cdot e^{-j\frac{2\pi f}{c}\left(d+d_{1}+d_{2}\right)}\cdot \rho_{\mathrm{ground}}\cdot \rho_{\mathrm{target}}}}+\underset{Path4}{\underset{︸}{\frac{1}{2d_{1}+2d_{2}}\cdot e^{-j\frac{2\pi f}{c}\left(2d_{1}+2d_{2}\right)}\cdot \rho_{\mathrm{target}}\cdot \rho_{\mathrm{ground}}^{2}}} \end{array}$$

The ratio between the total received power attained using the four-ray model and the power obtained from a single line of sight path is calculated by:

(5) $$\begin{array}{l} \frac{P_{r}\left(\mathrm{Four}\;\mathrm{rays}\right)}{P_{r}\left(\mathrm{LOS}\right)}=\frac{{\left|{\tilde{E}}_{Total}\right|}^{2}}{{\left|{\tilde{E}}_{LOS}\right|}^{2}}\\ ={\left|\;1+\frac{4d}{d+d_{1}+d_{2}}\cdot e^{-j\frac{2\pi f}{c}\left(d_{1}+d_{2}-d\right)}\cdot \rho_{\mathrm{ground}}+\frac{d}{d_{1}+d_{2}}\cdot e^{-j\frac{4\pi f}{c}\left(d_{1}+d_{2}-d\right)}\cdot \rho_{\mathrm{ground}}^{2}\right|}^{2}\\ ={\left|\;1+\left[\frac{4d}{d+d_{1}+d_{2}}+\frac{d}{d_{1}+d_{2}}\cdot \rho_{\mathrm{ground}}\cdot e^{-j\frac{2\pi f}{c}\left(d_{1}+d_{2}-d\right)}\right]\cdot \rho_{\mathrm{ground}}\cdot e^{-j\frac{2\pi f}{c}\left(d_{1}+d_{2}-d\right)}\right|}^{2} \end{array}$$

If the height *h*_radar_ of the radar antennas and the height *h*_target_ of the target above ground is substantially smaller than the distance, i.e., *h*_radar_, *h*_target_ << *d*, the total distance can be approximated by:

(6) $$d≅d_{1}+d_{2}-\frac{2h_{\mathrm{radar}}h_{\mathrm{target}}}{d_{1}+d_{2}}≅d_{1}+d_{2}-\frac{2h_{\mathrm{radar}}h_{\mathrm{target}}}{d}$$

Substituting the last expression into the power ratio (5), results in:

(7) Pr(Four rays)Pr(LOS)=|E˜Total|2|E˜LOS|2≅|1+(2+ρground⋅e−j4πfchradarhtargetd)⋅ρground⋅e−j4πfchradarhtargetd|2=1+4|ρground|2+|ρground|4+4[1+|ρground|2]Re{ρground⋅e−j4πfchradarhtargetd}+ 2Re{ρground2⋅e−j8πfchradarhtargetd}

An interesting case is that of grazing incidence, when the angle *θ*_0_ becomes close to 90 degrees. The reflection coefficients of both polarizations then approach:

(8) $$\rho_{\mathrm{ground}}≅\{\begin{array}{cc} -1=e^{j\pi } & TE\\ +1=e^{j0} & TM \end{array}$$

and Equation (7) can be approximated by:

(9) Pr(Four rays)Pr(LOS)=|E˜Total|2|E˜LOS|2≅6∓8Re{e−j4πfchradarhtargetd}+2Re{e−j8πfchradarhtargetd}=6∓8cos(4πfchradarhtargetd)+2cos(8πfchradarhtargetd)=4[1∓2cos(4πfchradarhtargetd)+cos2(4πfchradarhtargetd)]=4[1∓cos(4πfchradarhtargetd)]2={16sin4(2πfchradarhtargetd)TE16cos4(2πfchradarhtargetd)TM

The received power in the direct line-of-sight path is described by the radar formula [16]. Consequently, the total received power calculated using the four-ray model is:

(10) $$P_{r}\left(\mathrm{Four}\;\mathrm{rays}\right)≅G_{r}\cdot \underset{Path-loss}{\underset{︸}{\frac{\lambda^{2}}{{\left(4\pi \right)}^{3}d^{4}}\cdot \frac{P_{r}\left(\mathrm{Four}\;\mathrm{rays}\right)}{P_{r}\left(\mathrm{LOS}\right)}}}\cdot RCS\cdot \underset{EIRP}{\underset{︸}{G_{t}\cdot P_{t}}}$$

Inspection of the last derivation reveals that, for grazing incidence, the path loss of the vertical *TM* polarized radar link, grows in proportion to *d*^4^ (where *d* is the distance between the radar and the target), while for *TE* the path loss grows in proportion to *d*^8^.

## 4. Experimental Results

**Figure 4:**
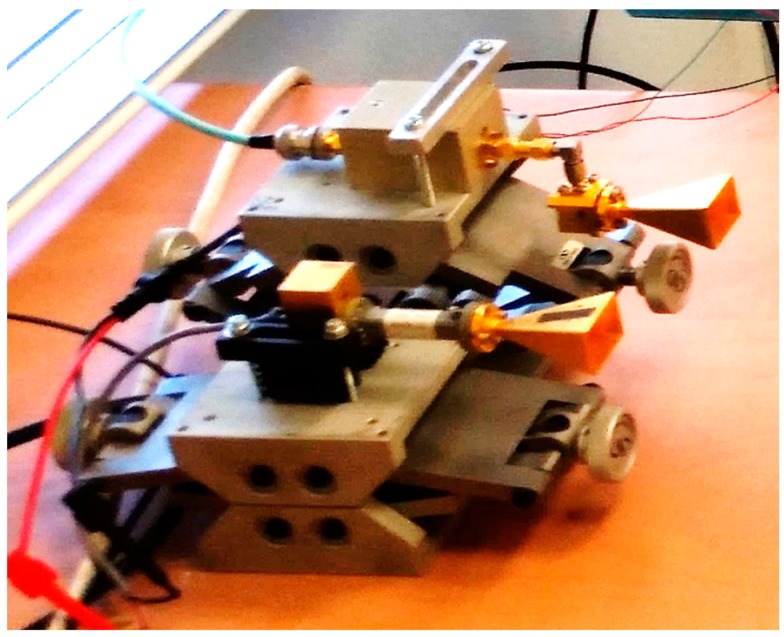
Multi-ray experiment setup. The transmitter and the receiver adjacent to each other.

The multi-ray effects were demonstrated using a short-range wireless link operating at millimeter wavelengths. A W-band transmitter and receiver were placed next to each other, with directional antennas looking at a target located on a motorized movable rail system. Due to the physical limitations of the system, the distances and heights were scaled down to imitate a radar operating on a vehicle. The antennas were placed at a height of 100 mm above a table, which served as a reflective surface, as shown in Figure 4. In order to ensure that the diffuse component in the scattering pattern is very small, a smooth table was used. The table roughness was well below the Rayleigh criterion *λ*/(8cos*θ*_0_) for all incident angles *θ*_0_ along the varying distance to the target (where *λ* = 3 mm at 94 GHz). Various objects were placed on top of a rod attached to the rail, serving as moving targets along the axial line. This experiment examined the effect of changing the distance between the transmitter and the scattering object, which reflects the signal to the receiver. Several types of reflecting object were used to examine their impact on the rays returned.

**Figure 5:**
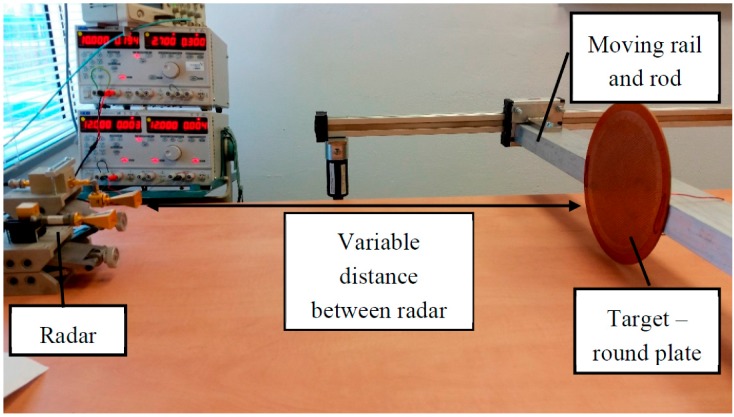
Multi-ray experiment with round plate.

The experiment was conducted at a frequency of 94 GHz. The transmitter was based on a Gunn diode, producing continuous power of 50 mW of constant envelope wave. The receiver was based on direct detection, with a diode serving as a square law detector. The voltage attained from the detector was amplified by a video amplifier with a gain of 10 dB in order to match the detected signal to a recording oscilloscope (see Figure 5). The typical bandwidth of the amplifier was about 50 kHz. The transmitting and receiving antennas were both horns, with a gain of 24 dBi, operating in the same vertical (TM) polarization.

In the first experiment, we demonstrated the reflection from a round plate. The plate diameter was 235 mm, and it was located close to the table, as shown in Figure 5. In this scenario, the plate formed a right angle with the table. This served as a specific reflector, causing only three possible ray paths between the transmitter and receiver.

**Figure 6:**
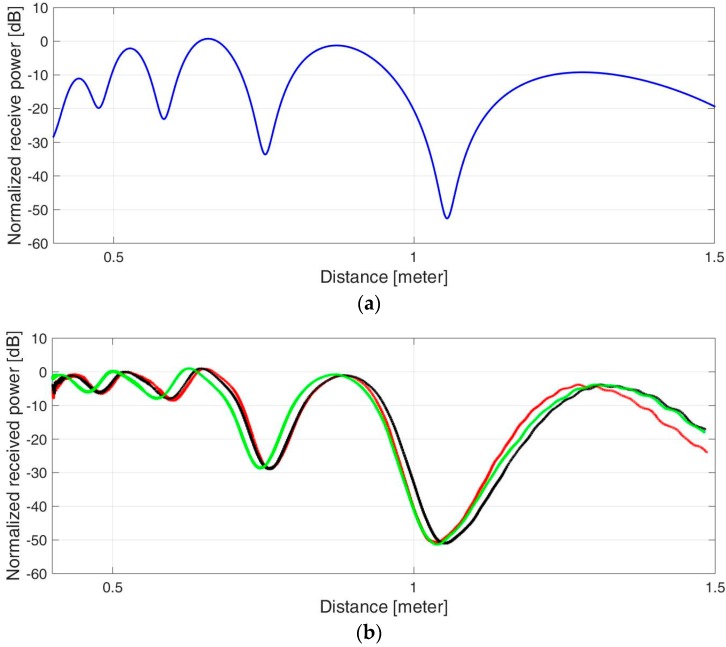
Received power in two-ray model: (**a**) Analytical calculation. (**b**) Experimental results for three similar experiments.

The experiment was carried out repeatedly, while the target moved on the radial axis back and forth between distances of 400–1500 mm. The received power was recorded, and graphs were drawn as a function of the distance. Figure 6 shows the normalized received signal strength vs. the distance to the target. A comparison between analytical calculations and experimental results was performed via the locations of peaks and dips of the signal strength measured along the distance, corresponding to constructive and destructive interferences. It can be seen that the power peaks and dips follow the two-ray model, where path 4 in Equation (4) is omitted. 

**Figure 7:**
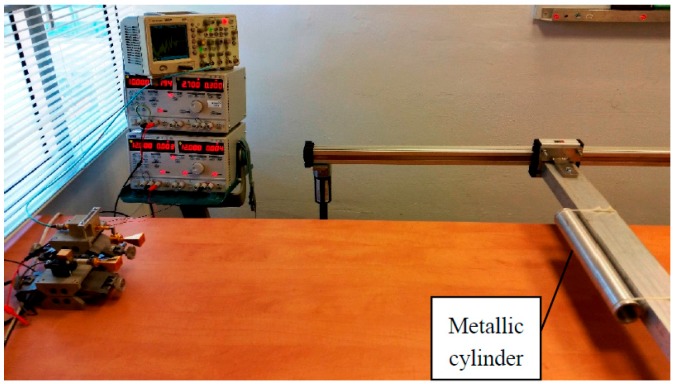
Multi-ray experiment with a metallic cylinder as a target.

A four-ray model was demonstrated by using a metal cylinder as a reflecting target. We used a 48 mm diameter cylinder with a length of 330 mm that was located 90 mm above the table (see Figure 7). As in the previous experiment, the object moved continuously in an axial line at distances between 400 mm and 1500 mm. In this case, the cylinder reflected the incident radiation at many angles, resulting in all four possible paths between the transmitter and receiver.

**Figure 8:**
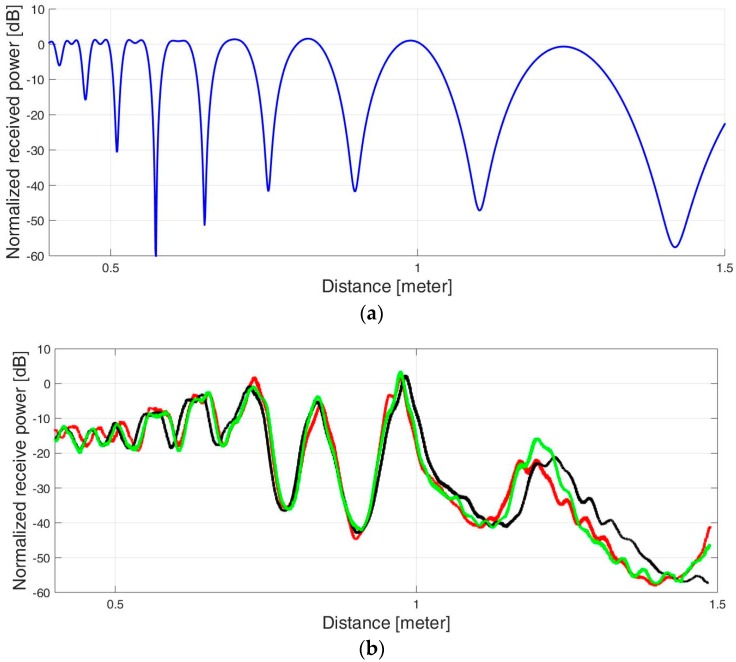
Four-ray model: (**a**) Analytical calculation. (**b**) Experimental results for three similar experiments.

The measured power was recorded and is presented in Figure 8. Inspection of the results reveals agreement between the theoretical four-ray model calculation and the experimental measurements.

## 5. Summary and Conclusions

This paper presents a multi-ray model for short-range radar, where reflections from the ground are involved. It is shown that, in general, there are four possible paths between the radar and the target that can be realized in a scenario where the radar is operating close to the ground (as in vehicles). The number of paths is determined by the reflective nature of the target. The multi-path causes peaks and dips in the received power as a function of distance. 

Experiments were conducted to verify the model. In our examinations, a continuous millimeter wave was transmitted towards different targets, using an automotive system. It was shown that the number of possible paths is determined by the target reflectivity. Two- and four-ray paths were demonstrated. 

## Figures and Tables

**Table 1:** W-band dielectric coefficients of materials.

Radar Material	Dielectric Constant ε′	Frequency
Soil (little moisture)	2.9	94 GHz
Soil (moist)	3.7	94 GHz
Acrylic 31	2.595	100 GHz
Ferroflow	13	100 GHz
Glass, Pyrex	4.33	100 GHz
Nylon	2.993	100 GHz
PE (Polyethylene)	2.306	100 GH
Asphalt	3.18	94 GHz
